# Extending Hospital-at-Home to nursing homes: findings from a novel care model in Singapore

**DOI:** 10.3389/fpubh.2025.1595535

**Published:** 2025-07-24

**Authors:** Chong Yau Ong, Angus Jun Jie Ng, Hui Juan Ngo, Eunice Jia Hwei Ya, Jean Mui Hua Lee

**Affiliations:** ^1^Sengkang General Hospital, Singapore, Singapore; ^2^SingHealth-Duke NUS Family Medicine Academic Clinical Program, Duke-NUS Medical School, Singapore, Singapore

**Keywords:** escalation, Hospital-at-Home, Hospital-at-Nursing Home, hospital-in-the-nursing home, length of stay, nursing home

## Abstract

**Background:**

We implemented a Hospital-at-Nursing Home (HaNH) pilot program in a nursing home to reduce acute hospital bed utilization and allow residents to receive right-sited care in familiar environments.

**Methods:**

A prospective data collection of the Hospital-at-Home (HaH) program was conducted from November 2023 to December 2024 in a regional general hospital.

**Result:**

16 HaNH enrollments were completed, comprising three admission avoidance cases and thirteen early supported discharges. Pneumonia (56.3%) and urinary tract infections (18.8%) were the most common diagnoses. The median length of stay was three days (range 1–12, IQR 4). One mortality occurred within the program in the HaNH in alignment with the patient’s preferred place of care and death, supported by palliative care. Comparisons with a non-institutionalized HaH cohort (*n* = 349) had a higher risk of escalation to the actual hospital facility (RR = 5.45, 95% CI: 1.71–17.42, *p* = 0.0025; aRR = 1.32, 95% CI: 0.35–4.96). HaNH patients had increased vulnerability, with higher post-discharge mortality (RR = 10.9, 95% CI: 2.16–55.21, *p* = 0.004; aRR = 3.38, 95% CI: 0.83–13.71) and emergency visits (RR = 3.18, 95% CI: 1.72–5.88, *p* = 0.0002; aRR = 2.00, 95% CI: 1.18–3.36), though readmission risk was non-significant.

**Conclusion:**

These preliminary findings suggest that while HaNH may alleviate hospital bed shortages, patients in nursing homes are at increased risk of deterioration and require careful selection and support.

## Introduction

Even with the development of home-based services and assisted living facilities, the institutionalization of older adults remains prevalent in a rapidly aging population (1). Nursing home (NH) residents often present with multiple comorbidities, cognitive impairment, frailty, and disabilities, making them more susceptible to acute illnesses that require hospitalization. Compared to community dwellers, nursing home residents experience longer hospital stays and a higher risk of functional decline (2). The average hospital length of stay for nursing home residents in Singapore is 8 days, surpassing the national average of 5 days (3). Frequent hospitalizations contribute to increased healthcare resource utilization without significant prognostic improvement, necessitating alternative care models.

Hospital-at-Home (HaH) is a growing alternative care model to address the burgeoning need for healthcare resources (4). In the land-scarce city-state of Singapore, building more brick-and-mortar infrastructure may not be sustainable (5). For our hospital in 2023, the percentage of healthcare utilization of the number of all older adults (aged 65 and above) was 36.1% in the outpatient setting. While 31.1% of the patients seeking healthcare from the emergency department were older adults, the proportion of older adults who were hospitalized was higher at 56.3%. This amassed to 66.8% of the bed days, attributed to all older adults who were hospitalized in our hospital. HaH allows patients to receive inpatient-level care in their homes. From the hospital’s perspective, a reduction in the use of hospital beds is achieved through the complete substitution of ward stay in admission avoidance when the patients are enrolled from the emergency department or through early supported discharge after the patients are stabilized in the first few days in the hospital (6, 7). HaH has been associated with the optimization of hospital facility without compromising clinical outcomes (8, 9), as well as higher level of patient satisfaction (10, 11).

Although HaH has been well established in other countries, its implementation in Singapore and a large part of Asia remains in its infancy. The HaH program was introduced in Singapore in 2018, with expanded implementation during the COVID-19 pandemic, similar to other parts of the world (12, 13). This model offers hospital-level care in patients’ homes through remote monitoring, medication administration, and virtual clinical consultations (14).

Recognizing the benefits of HaH, the Ministry of Health approved the Hospital-at-Nursing Home (HaNH) pilot to extend acute care delivery to one selected nursing home. This represents a pioneering care model in Singapore and Asia. Hospital-in-the-nursing-home has been implemented in Australia for more than a decade as an expansion of its hospital-in-the home service (15, 16). The program’s doctors and or nurses would do a physical site visit if necessary, on top of the daily morning reviews via teleconsultation. For any queries, patient may use messaging platforms to contact the doctors. Similarly, the program’s doctors would also update the patient and next-of-kin when investigation results are completed. The partnered nursing home also has a resident doctor who is present once to thrice weekly. The program’s value proposition lies in admission avoidance and early supported discharge, ensuring that nursing home residents receive hospital-level care within their residence while aligning with value-based care principles. By receiving acute-level care in the nursing home, nursing home residents can also avoid hospital-acquired infections which commonly occur among older adult patients. We seek to compare the post -discharge outcomes (ED visits, hospitalisation, and mortality) with the prevailing HaH to assess the feasibility of HaNH to reduce the demand of inpatient beds.

**Figure 1:**
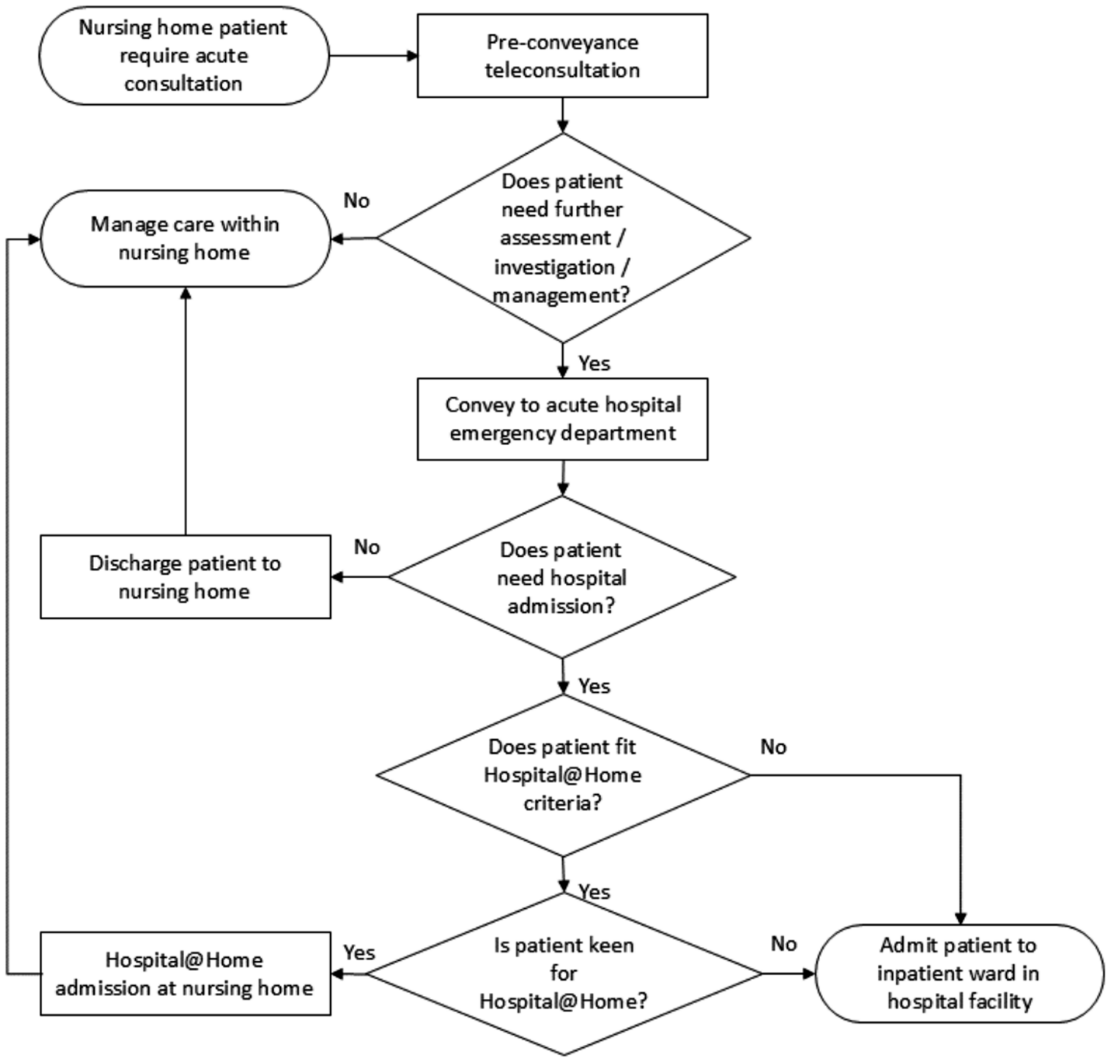
Workflow for HaNH to access need of hospital-level care, need for hospitalization, and suitability and keenness for the HaNH.

Patients were referred to HaNH through primary clinical teams from emergency departments or inpatient wards. Suitability assessments determined eligibility for admission avoidance or early supported discharge (Supplementary Table 1). The scope of HaNH included diagnosis confirmation stabilization, antibiotic completion, biochemical monitoring, and palliative care where applicable. The workflow of patient transition into the HaNH program is illustrated in Figure 1.

## Methods

### Study design

This is a prospective study of outcomes of 349 and 16 patients enrolled in the Hospital-at-Home and Hospital-at-Nursing Home, respectively, in a regional hospital in Singapore. Enrollments between November 11, 2023 (implementation of HaNH) and December 31, 2024 were included and compared.

### Outcomes

We compared the outcomes during the program (mortality, length of stay and rate of escalation to actual brick-and-mortar hospital facility), and post-discharge events (mortality, emergency department visit, and non-elective readmissions) over a timeline of 30-day, 60-day, and 90-day, respectively. The length of stay refers to the number of days the patient spent in the HaH and HaNH programs respectively, excluding the time in the emergency department and actual hospital facility.

### Statistical analysis

Data were collected from a tertiary general hospital’s HaH and HaNH programs, with descriptive statistics reported. The analysis was performed using Microsoft Excel (M365, Redmond, WA). Mann–Whitney U-test and Chi-square test were employed to compare the clinical outcomes of length of stay and escalation to actual hospital facility, respectively. Post-discharge events were reported as relative risks with confidence intervals. Adjusted relative risks were calculated using Poison regression with age and gender corrections using Python (PFS, DE).

### Ethical approval

This study was reviewed by the SingHealth Centralised Review Board and decisioned to be not requiring review (CIRB: 2024/2252).

## Results

Sixteen patients were enrolled in HaNH, with three cases recruited directly from the emergency department (admission avoidance) and thirteen from the inpatient wards as early supported discharge. The leading admission diagnoses were pneumonia (56.3%) and pyelonephritis (18.75%). Table 1 summarizes the demographic characteristics of HaNH and HaH patients.

**Table 1:** Demographics of patients in Hospital-at-Nursing Home and Hospital-at-Home.

	Hospital-at-Nursing Home (*n* = 16)	Hospital-at-Home (*n* = 349)
Age (mean, ± SD)	80.3 ± 11.3	54.5 ± 20.8
Gender *n*, (%)		
Female	3 (18.8)	150 (43.0)
Male	13 (81.3)	199 (57.0)
Enrolment source		
Admission avoidance*	3 (18.8)	136 (38.9)
Early supported discharge	13 (81.3)	213 (61.0)
Diagnosis *n*, (%)		
Pneumonia	9 (56.3)	34 (9.7)
Pyelonephritis and UTI	3 (18.8)	60 (17.2)
Cellulitis	1 (6.3)	57 (16.3)
Others	3 (18.8)	131 (37.5)
Dengue		36 (10.3)
Rhabdomyolysis		19 (5.4)
Gastroenteritis		12 (3.4)

^*^Includes emergency department, emergency department observation, and specialist outpatient clinic. UTI: urinary tract infection.

The median length of stay was 3 days (range 1 to 12, IQR 4), comparable to HaH patients (*z* = 0.34, *p* = 0.73). This suggests that HaNH can provide timely acute care without prolonging the duration of illness. One mortality occurred within HaNH under palliative care, aligning with the patient’s preferred place of care and death. No in-program mortality was observed in the HaH cohort.

Three escalations to actual hospital facility from HaNH were due to deterioration in clinical conditions from supraventricular tachycardia, sepsis unresponsive to fluid boluses, and desaturation. HaNH patients had significantly higher escalation to actual hospital facility 18.8% vs. 3.4%, (*χ*^2^ = 9.10, *p* = 0.0025), with an unadjusted RR of 5.45 (95% CI: 1.71 to 17.42) (Table 2). After adjusting for age and gender, the risk was attenuated (aRR = 1.32, 95% CI: 0.35 to 4.96, *p* = 0.68). The adjusted aRR of 1.32 suggests that while age and gender contributed to this disparity, other factors such as underlying functional status and resource constraints in nursing homes can be improved.

**Table 2:** Comparison of outcomes during program (inpatient-level at nursing home vs. inpatient at home).

	HaNH	HaH	*p* value
Length of stay (days), median [Q_1_, Q_3_], range	3 [1.5, 5.5], range 1–12	3 [1, 2], range 1–21	0.73
Escalation to actual hospital facility, *n* (%)	3 (18.8)	12 (3.4)	0.00025*

*aRR reported in Table 4.

**Figure 2:**
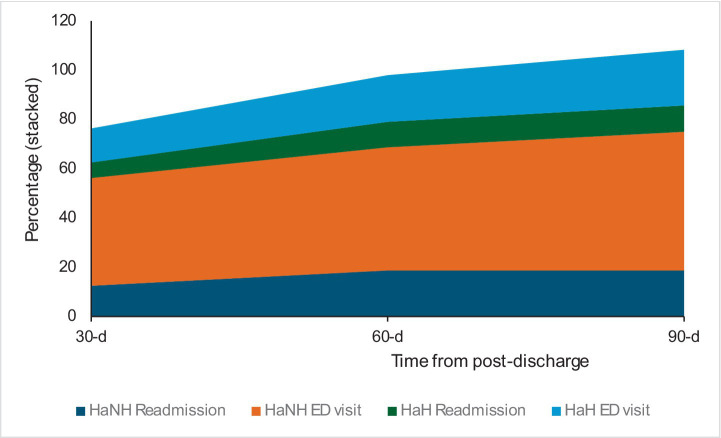
The stacked area chart showed a high proportion of post-discharge ED visits from the HaNH cohort compared to HaH consistently across three periods.

Post-discharge outcomes were consistently worse for HaNH patients (Table 3). The 30-day post-discharge mortality risk was significantly higher (RR = 10.9, 95% CI: 2.16–55.21, *p* = 0.004), though this declined at 60 days (RR = 9.35, 95% CI: 2.66–32.83, *p* = 0.0005) and 90 days (RR = 7.27, 95% CI: 2.18–24.30, *p* = 0.001). A similar trend was observed for emergency department visits, with the highest risk at 30 days (RR = 3.18, 95% CI: 1.72–5.88, *p* = 0.0002), decreasing at 60 days (RR = 2.64, 95% CI: 1.55–4.52, *p* = 0.0004) and 90 days (RR = 2.48, 95% CI: 1.55–4.52). This is expected, given the greater frailty and lower physiological reserves of nursing home residents. Readmission rates followed a comparable pattern but were not statistically significant. Figure 2 shows the stacked percentages of HaNH readmissions, HaNH ED visits, HaH readmissions, and HaH ED visits across the three post-discharge timelines. Adjusted analyses showed aRRs of 3.38 (95% CI: 0.83–13.71) for mortality, 2.00 (95% CI: 1.18–3.36) for emergency visits, and 1.12 (95% CI: 0.47–2.68) for readmissions (Table 4).

**Table 3:** Comparison of post-discharge outcomes (all numbers in percentages).

Rates (%)	HaNH			HaH		
	Readmission	ED visit	Mortality	Readmission	ED visit	Mortality
30-d	12.5	43.75	12.5	6.3	13.75	1.15
60-d	18.75	50.0	18.75	10.3	18.9	2.01
90-d	18.75	56.25	18.75	10.7	22.6	2.58

**Table 4:** Adjusted relative risks (aRR) for the outcome measures.

Outcomes	aRR	95% CI
Within program		
Escalation to hospital facility	1.32	0.35–4.96
Post-discharge		
Mortality	3.382	0.83–13.71
Readmission	1.126	0.47–2.68
ED visit	1.995	1.18–3.36

## Discussion

To our knowledge, this is the first implementation and comparison of the clinical outcomes of Hospital-at-Nursing Home (HaNH) with those of Hospital-at-Home (HaH). Our findings suggest that HaNH is a feasible alternative care model for managing acute medical conditions among nursing home residents, achieving comparable lengths of stay to Hospital-at-Home (HaH). However, the higher risk of escalation to an actual hospital facility highlights the need for careful patient selection and tight clinical partnerships with nursing home care teams to provide robust clinical support.

The predominance of pneumonia as a leading admission diagnosis is consistent with global trends, as pneumonia remains a major cause of hospitalization and mortality in older adults (17, 18). Current studies demonstrated safety and efficient care in treating pneumonia in the HaNH setting when compared to treating them in the actual hospital facility (19, 20); our results highlight the importance of close monitoring and early deterioration detection.

The median length of stay for HaNH patients was 3 days, which did not differ significantly from that of HaH patients. Previous studies on HaH have emphasized its role in reducing hospital bed occupancy while maintaining clinical safety (21), and our findings extend this understanding to the nursing home setting. Traditionally, nursing home residents are frequently conveyed to the hospital for diagnostic work-up, medical treatment, and palliative and end-of-life supportive care (22). HaNH ameliorates the need for hospitalization in a brick-and-mortar hospital facility for the above aims.

Despite similar lengths of stay, HaNH patients had a significantly higher rate of escalation to a hospital facility (18.8% vs. 3.4%, *p* = 0.0025). This is likely due to frailty, multimorbidity, and the inherent limitations of delivering hospital-level care in nursing homes.[23, 24]Given that pneumonia and urinary tract infections were the most common diagnoses, close monitoring and early recognition of clinical deterioration are critical in HaNH programs (23). Diagnostic and therapeutic resources are more constrained in this setting due to inherent limitations. Future research should investigate whether targeted interventions, such as enhanced telemonitoring, onsite diagnostics, or modified inclusion criteria using predictive modeling, could mitigate escalation risks (24, 25).

One mortality occurred in the HaNH group under palliative care, which was aligned with the patient’s preferred place of care and death. One systematic review meta-analysis showed HaH probably makes little or no difference on mortality at 6 months follow-up (26). However, there is a huge potential for HaNH to support goal-concordant care, particularly for patients with advanced illness who may benefit from acute symptom management in a familiar setting without the distress of hospital transfer (27).

Post-discharge, HaNH patients demonstrated consistently worse outcomes, including higher mortality rates and emergency department visits, particularly in the first 30 days. Notably, mortality risk declined over time, suggesting that early post-discharge interventions may improve outcomes (28). This is in keeping with prior research showing that nursing home residents remain vulnerable to adverse events even after hospitalizations (29–31).

Despite a higher risk of escalation back to the hospital facility during course of the program and higher post-discharge events, the HaNH pilot program demonstrated feasibility and safety in delivering acute care within nursing home settings. One major benefit includes maintaining residents in familiar environments, thus reducing delirium risk and improving staff satisfaction through increased clinical involvement (14). Another benefit of reducing inpatient stay would be to reduce the possibility of contracting hospital-acquired illnesses which may prolong their hospital stay, leading to increased hospital utilization (32). Proximity of medical service plays an important role in the decision of preferred place of care in the older adults (33). With the HaNH, the residents can be managed in their nursing homes.

Some of the challenges included extensive logistical coordination and increased physician time commitment compared to traditional inpatient care (34). Most nursing homes already have in-house or outsourced general practitioners (GPs) arrangement, thus, the HaNH team needed to effectively coordinate care with these GPs to ensure seamless patient management. This was done tactfully while navigating governance complexities, liability concerns, and duty of care obligations. A collaboration agreement was established between HaNH and the nursing home to align clinical responsibilities, clarify professional accountability, and mitigate medicolegal risks. The other challenge was increased workload on nursing home staff when acutely ill patients are being cared in the nursing home settings. This would demand more attention, monitoring, and vigilance among the nursing home staff. If not managed well, it might divert resources away from routine care provision to the other residents. Balancing these competing care priorities would be an operational challenge. However, these were mitigated through tight coordination using mutually agreed workflows and structured communication channels, including email and secure text communication portals.

This study has several limitations. The relatively small sample size in the HaNH group limits the generalizability of our findings. This would most likely lead to type 2 error in detecting a real effect. Of eligible patients admitted from the one nursing home, only about 65% were successfully enrolled into HaNH. Implementing HaNH was contingent on the acceptability of this ‘novel’ care among family members of the residents. In a discrete choice experiment study, family members were found to be less tolerant of care quality reduction delivered to the residents than the resident and nursing home staff (35). The perception of the brick-and-mortar hospital facility as the best place of care regardless of presenting condition has been ingrained in the public and would need time to be changed. To address this, extensive engagement efforts emphasized that HaNH provides equivalent medical oversight while prioritizing patient comfort and preferences. The time-intensive nature of promoting the HaNH as a mainstream service highlighted the importance of clear communication in fostering public acceptance. Future prospective studies with larger sample sizes are needed to confirm our findings and further elucidate the role of HaNH in different healthcare settings.

Secondly, cost-effectiveness analyses should be undertaken to ascertain the tangible benefits to the healthcare system (hospitals and nursing homes) and the nursing home residents. This would involve direct and indirect costs. Direct costs include manpower, medical supplies and equipment, management costs (for procedures and treatment done by hospital and vendor nurses) and facility costs. Indirect costs include transportation costs, training, and capacity building. This should be measured against potential savings from emergency department visits, acute hospitalization avoidances and reduction of complications of hospitalization.

## Conclusion

Our study provides preliminary evidence supporting the feasibility of HaNH as an alternative model of acute care delivery for nursing home residents. While HaNH achieved similar lengths of stay to HaH, the significantly higher escalation rate highlights the need for clinical and delivery improvements. Future research should explore strategies to explore scalability, optimize patient selection, improve monitoring capabilities, evaluate long-term patient outcomes, and lay strategies to better engage caregivers in HaNH programs. This would inform policymaking and model of care use such as expansion to more nursing homes nationwide and strengthened advanced care planning and end-of-life care implementation following directives of the Ministry of Health.

## Data Availability

The raw data supporting the conclusions of this article will be made available by the authors, without undue reservation.
