# Supplementary material for: Extending Hospital-at-Home to nursing homes: findings from a novel care model in Singapore
Source: Front Public Health. 2025 Jul 24;13:1595535. doi: 10.3389/fpubh.2025.1595535 (PMC12328139; doi:10.3389/fpubh.2025.1595535)
Supplement: Supplementary file 1 [file Table_1.docx]

Supplementary Table 1: Criteria to Hospital-at-Home

| General inclusion criteria | General exclusion criteria |
| --- | --- |
| Stable vital signs.  Able to self-care or have caregiver support.  Have internet or data for connectivity for telemonitoring. | Unstable vital signs.  Requiring telemonitoring more than 6-hourly interval.  Suicidal ideation. |
| Eligible conditions (non-exhaustive) | |
| Cellulitis  Dengue  Gastroenteritis  Gout  Heart failure  Hyperglycemia not in crisis  Intra-abdominal abscess  Liver abscess  Lower back pain  Rhabdomyolysis  Pneumonia  Post-bariatric surgery  Post-minimally invasive surgery  Prostatic abscess  Pyelonephritis  Palliative care needs  Urinary tract infections | |

*Condition-specific exclusions:

For cellulitis: able to be managed outpatient with oral antibiotics, suspicion of necrotizing infection needing surgery

For dengue: severe dengue with significant bleeding manifestation, third space loss, or severe organ involvement.

For heart failure: Has a left ventricular assist device, unable to accurately chart urine output at home, requiring oxygen therapy, significant acute renal impairment Cr>50% baseline, K<3.0

For hyperglycemia not in crisis: evidence of diabetes ketoacidosis (DKA), hyperosmolar hyperglycemia syndrome (HHS), needing sliding scale insulin, on insulin pump, brittle diabetes.

For liver abscess: Requiring daily flushing of drains

For pneumonia: Most recent CURB-65 score >3, cavitary lesion on imaging, pulmonary effusion of unknown etiology, requiring oxygen support.

For post minimally invasive surgery and post bariatric surgery: requiring daily review by surgical team, suspicion of surgical complication requiring another surgical intervention

For pyelonephritis: pregnant woman with pyelonephritis, obstructive pyelonephritis, demonstrable pyonephrosis, clinically well and able to be treated with oral antibiotics.

For rhabdomyolysis: creatine kinase less than 40,000. McMahon score> 60
